# The Test-Retest Reliability of New Generation Power Indices of Wingate All-Out Test

**DOI:** 10.3390/sports6020031

**Published:** 2018-04-07

**Authors:** Ozgur Ozkaya, Gorkem Aybars Balci, Hakan As, Emre Vardarli

**Affiliations:** 1 Coaching Education Department, Faculty of Sport Sciences, Ege University, Izmir 35050, Turkey; gorkem.aybars.balci@ege.edu.tr (G.A.B.); emrevardarli@hotmail.com (E.V.); 2 Health Sciences Institution, Ege University, Izmir 35050, Turkey; hakanas@windowslive.com (H.A.)

**Keywords:** anaerobic, instantaneous, maximum speed, rpm, time to peak power

## Abstract

Although reliability correlations of traditional power indices of the Wingate test have been well documented, no study has analyzed new generation power indices based on milliseconds obtained from a Peak Bike. The purpose of this study was to investigate the retest reliability of new generation power indices. Thirty-two well-trained male athletes who were specialized in basketball, football, tennis, or track and field volunteered to take part in the study (age: 24.3 ± 2.2 years; body mass: 77 ± 8.3 kg; height: 180.3 ± 6.3 cm). Participants performed two Wingate all-out sessions on two separate days. Intra-class correlation coefficient (ICC), standard error measurement (SEM), smallest real differences (SRD) and coefficient of variation (CV) scores were analyzed based on the test and retest data. Reliability results of traditional power indices calculated based on 5-s means such as peak power, average power, power drop, and fatigue index ratio were similar with the previous findings in literature (ICC ≥ 0.94; CV ≤ 2.8%; SEM ≤ 12.28; SRD% ≤ 7.7%). New generation power indices such as peak power, average power, lowest power, power drop, fatigue index, power decline, maximum speed as rpm, and amount of total energy expenditure demonstrated high reliability (ICC ≥ 0.94; CV ≤ 4.3%; SEM ≤ 10.36; SRD% ≤ 8.8%). Time to peak power, time at maximum speed, and power at maximum speed showed a moderate level of reliability (ICC ≥ 0.73; CV ≤ 8.9%; SEM ≤ 63.01; SRD% ≤ 22.4%). The results of this study indicate that reliability correlations and SRD% of new generation power and fatigue-related indices are similar with traditional 5-s means. However, new time-related indices are very sensitive and moderately reliable.

## 1. Introduction

The Wingate all-out test requires pedaling with maximal effort for 30 s against a constant workload that is based on body mass [1]. The test is easy to administer, non-invasive, and can be conducted with an inexpensive mechanically braked cycle ergometer, thus it has achieved widespread use among laboratories for analyzing the anaerobic performance of various population groups [2,3,4,5,6,7]. Peak Power (PP_5s_), Average Power (AP_5s_), Lowest Power (LP_5s_), Power Drop (PD_5s_), and Fatigue Index (FI_5s_) have been well-known traditional power indices [1]. PP_5s_, AP_5s_, and LP_5s_ are calculated over averages of 5-s intervals. FI_5s_ and PD_5s_ are the fatigue-related power indices and they are calculated via PP_5s_ and LP_5s_. PD_5s_ is the power drop-off throughout the test, while FI_5s_ is the power decline as a percentage of the PP_5s_.

In the 2000s, mechanically braked new Peak Bike 894E ergometers with hardware and software upgrades were introduced. Nowadays, it is possible to obtain new generation power indices which are calculated in millisecond sensitivity instead of 5-s means (14/52/6= Ratio flywheelto crank / 6 magnets). The new generation power indices include instantaneous outcomes such as Peak Power (PP) as the highest running average of one second in watts; Average Power (AP) as the average power of the entire test in watts; Lowest Power (LP) as the lowest running average of one second period in watts; Fatigue Index (FI) as relative power decline from start to end; Power Drop (PD) as drop in power from start to end. Additionally, it is possible to obtain new parameters such as time to peak power (t_pp_) as time to elicit peak power from start to middle of the one second period in milliseconds; maximum speed as rpm (*v*_max_) reached maximum instantaneous speed during the whole test as rpm; power at maximum speed (P@*v*_max_) as power at time of maximum rpm; time at maximum speed (t@*v*_max_) as the time at maximum speed in milliseconds; decline in power (P_dec_) as the difference between peak power and power at the end; and amount of total energy expenditure (*e*_tot_). It can be said that PP, t_pp_, *v*_max_, P@*v*_max_, and t@*v*_max_ are the measures of explosive power (alactic performance, neuromuscular fatigue, etc.), while AP, LP, FI, PD, P_dec_, and *e*_tot_ are used to measure lactic (anaerobic) capacity. 

Numerous studies have reported that the traditional power indices have high reliability correlations ranging from 0.89 to 0.98 [1,8,9,10,11]. The validity of the traditional power indices has been examined by correlating them with field and laboratory test results. Observations showed that validity correlations have a wide range that reaches up to 0.92 [5,12,13,14,15]. However, the validity of Wingate’s traditional indices tend to have low correlation coefficients (r = 0.32) when using skill specific test results as criterion [16]. Although retest reliability and the validity of traditional power indices have been well documented since the end of 1970s, reliability analyses of new generation indices have not, to the authors’ knowledge, been analyzed yet. The new generation indices can sensitively determine and classify athletic performance due to both an increasing number of new parameters and their sensitivities. As such, nowadays, new generation indices are preferentially used for standard laboratory assessments and scientific texts instead of traditional 5-s means. However, there has been only one study, reported by Jaafar et al. [17], that has evaluated effects of different work-loads (8.7 and 11% of body mass) on the reliability of the Wingate test indices and some physiological responses. Intra-class correlation coefficient (ICC) scores of the test indices were reported as greater than the level of 0.89 in this study. However, the effects of traditional Wingate test load (10% of body mass for trained athletes [1,18]) and reliability correlations of the new generation power outputs such as t@*v*_max_, P@*v*_max_, t_pp_, *v*_max_, and *e*_tot_ were not analyzed in this study. The aim of this study was therefore to determine the retest reliability of both traditional and new generation power, fatigue, and time-related indices obtained from the Wingate all-out test by using the recommended traditional work-load of 10% of body mass.

## 2. Materials and Methods

### 2.1. Experimental Approach to the Problem

A repeated measures study design was carried out in this study. Participants performed comprehensive familiarization sessions in order to overcome the learning effect. Subsequently, two Wingate all-out trials were conducted on two separate days for data collection (Session-1 and Session-2). The results were used to investigate the test-retest reliability. The testing time of day was standardized for each session to minimize any effects of circadian variance. Additionally, test sessions were conducted in a laboratory in order to provide standard climatic environment (20–21 °C temperature and 50–55% relative humidity) for each session.

### 2.2. Participants

Thirty-two well-trained male athletes who did not have any serious injury background volunteered to take part in the study (age: 24.3 ± 2.2 years; body mass: 77 ± 8.3 kg; height: 180.3 ± 6.3 cm). Athletes were specialized in basketball, football, tennis, or track and field. They were participating in physical training for about 6–8 h∙wk^−1^ throughout the test period. Written informed consent for participation was obtained after the explanation of the purpose and the test protocol. The approval of the university ethics committee was also granted (EGE.SBF.16-04/11). Participants were requested not to take part in any exhaustive exercise 24 h prior to the testing sessions. They were also asked not to take any beverages containing alcohol or caffeine a day before the test.

### 2.3. Procedures

Familiarization sessions and main tests were performed on a mechanically braked Monark Peak Bike cycle ergometer (Peak Bike 894, Monark, Vansbro, Sweden). Seat height and handle bar adjustments were made for each participant to allow for a comfortable riding position. Additionally, toe clips were used to allow participants to apply correct pedaling techniques [19]. They were instructed to preserve their seating position throughout sessions to avoid any effect of postural change [20].

### 2.4. Familiarization Sessions

The aim of the familiarization sessions was to adapt participants to the Wingate protocol and test specificities. Familiarizing session was consisted of four stages: (*i*) Since a correct pedaling technique is important to reveal potential maximum power production [21], participants performed pedaling exercises for two sets of 10 min each [22]. (*ii*) Then, flywheel’s rope was taken off and Peak Bike was used as a type of Power Cycle [23]. We asked participants to accelerate the flywheel and reach maximum velocity as soon as possible. When they achieved the score of less than two seconds at the end of two successive bursts, they upgraded to the next level of familiarization process. (*iii*) After enough time to rest, we asked the participants to experience just the beginning of the Wingate all-out test administration (5–6 s). After an initial burst of a 2–3-s unloaded period, a test-load, corresponding to 10% of body mass, was applied to the system [1,18] at 120 rpm [23,24,25,26,27,28,29]. Participants tried to reach time to peak power less than two seconds. We carried out repetitions until participants could overcome two successive bursts. (*iv*) After enough time for recovery (a period of 5–6 h in general), participants performed a single 30-s all-out test to gain practice effect which is a necessity before the real test administration [30]. Strong verbal encouragements were given throughout the administration. Additionally, we questioned Borg’s scale (6 to 20) and checked whether participants gave the scores of 19 or 20 [31]. Moreover, we analyzed the power-time curve to detect any segmental disorder [32]. If there was a gradual decrement in power-time curve and there was no any error, participants become entitled to take part in the main reliability study.

### 2.5. Wingate All-Out Test Administrations

After a period of 24 h, the first test (session-1) was performed. Each participants cycled at 70–80 rpm against a workload corresponding to 2% of his individual body mass as a warm-up. In this period, they performed three bursts of 3 s at the end of the third, fourth, and fifth minute. Then, participants rested for 5 min following the warm-up session [18]. The test-load was set at 10% of each individual’s body mass, as recommended for trained male athletes. Participants started the all-out test administration with maximal effort without a load until they reached 120 rpm to overcome the flywheel inertia. Then, the test-load was applied to the system, and data collection was immediately commenced. Strong verbal encouragements were given throughout the test. At the end of the all-out administration, athletes were asked to cycle for another 5 min for gradual cool down. The next day, participants performed second tests (session-2) by the same standards.

### 2.6. Statistical Analysis

Data were evaluated by SPSS 21 (SPSS Inc., Chicago, IL, USA). Levene’s test was used to check variance homogeneity. The Shapiro-Wilk test was used for determining normality of data distribution. Relative reliability was analyzed by the intra-class correlation coefficient (ICC) and 95% confidence interval (CI) was estimated with a two-factor mixed-effect model with absolute agreement. Absolute reliability was assessed by standard error measurement (SEM), which is calculated by dividing the standard deviation of difference with $\sqrt{2}$ [16]. Smallest real differences (SRD) were calculated as $1.92\times SEM\times \sqrt{2}$. SRD% was calculated by dividing SRD by the mean of two tests multiplied by 100 [33]. The agreements between measurements were verified qualitatively using Bland and Altman plots [34]. SEM and mean values in both tests were used in order to compute coefficient variation (CV) [35]. Smallest worthwhile change (SWC) was determined by multiplying between-subjects standard deviations (SD) with 0.2. Effect size values were determined by Cohen’s d. Results that were 0.2–0.49 were considered “small”, 0.5–0.79 “medium”, and ≥0.8 “large”. The statistical significance level was set at *p* ≤ 0.05 for all analyses.

## 3. Results

The results of test-retest reliability of traditional power indices are displayed in Table 1. Among traditional Wingate indices, PP_5s_ showed the highest reliability coefficients, as expected (ICC = 0.99; CV = 0.97%; SRD% = 2.69%). 

The test-retest reliability correlations of new generation power indices are presented in Table 2. PP, AP, LP, PD, FI, *v*_max_, P_dec_, and *e*_tot_ showed high reliability (ICC ≥ 0.94; CV ≤ 4.3%; SRD% ≤ 8.78%). t_pp_, P@*v*_max_, and t@*v*_max_ demonstrated moderate reliability coefficients (ICC ≥ 0.73; CV ≤ 8.9%; SRD% ≤ 22.43%).

**Figure 1:**
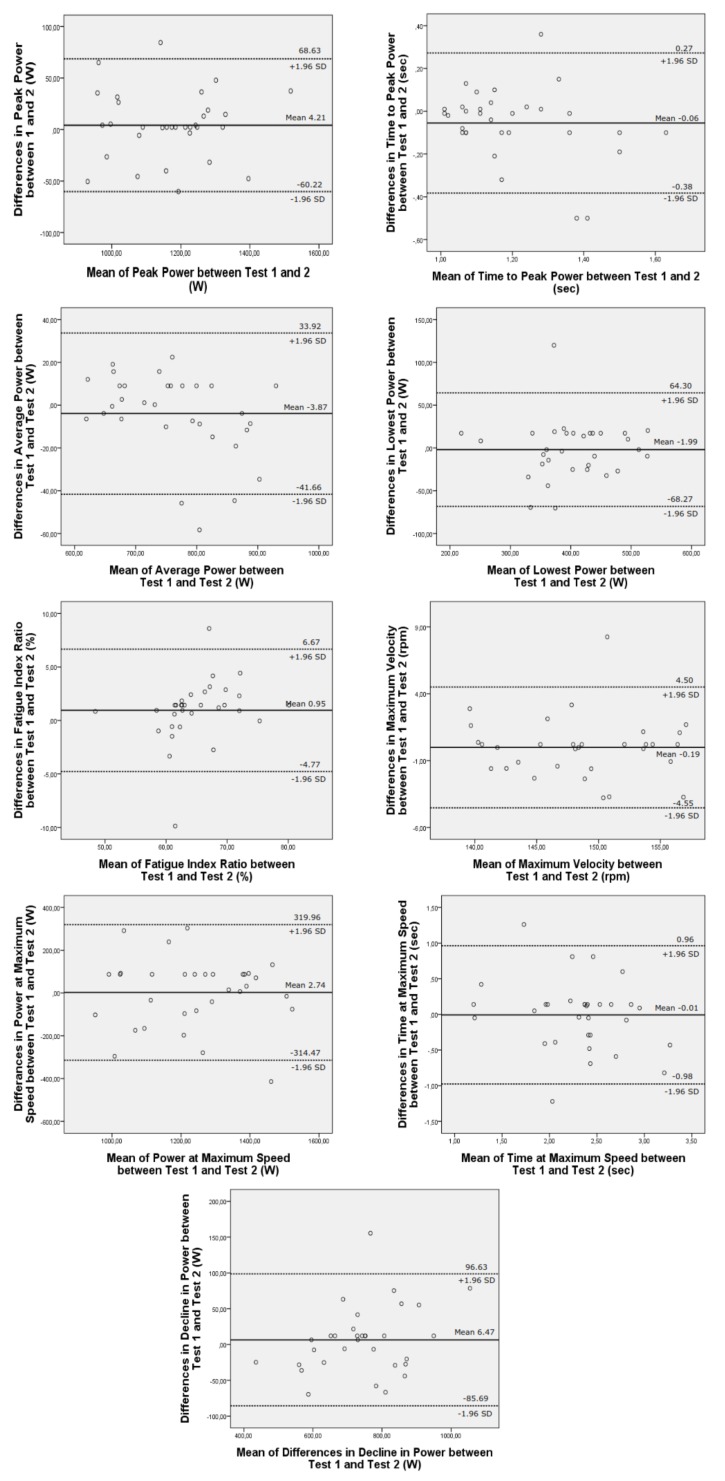
Bland-Altman plots showing test-retest reliability of new generation power indices (*n* = 32).

Bland-Altman plots for mean new generation power indices (Figure 1) illustrates that no major systematic bias was found in all plots, although there were some outliers. Analyses also revealed small ratio effects and heteroscedasticity in differences between repeated trails. 

## 4. Discussion

Since new generation indices can sensitively determine and classify athletic performance due to both an increasing number of new parameters and their sensitivities based on raw data, new generation indices means have been increasingly used for standard laboratory assessments and scientific texts instead of tradition 5-s means. However, as of yet, there has not been a comprehensive reliability study in this regard. The aim of this study was therefore to investigate the retest reliability of the new generation power indices. In the present study, traditional indices were found to have high ICC values, and low scores for both SEM and SRD%. Moreover, reliability levels of PP, AP, LP, PD, FI, *v*_max_, P_dec_, and *e*_tot_ were found to be reliable and similar with the traditional ones (ICC ≥ 0.94; score of SRD% ≤ 8.78%). However, power and time-related indices calculated based on milliseconds such as t_pp_ (ICC = 0.73; CV = 4.53%; SRD% = 12.56%), P@*v*_max_ (ICC = 0.76; CV = 6.55%; SRD% = 14.63%), and t@*v*_max_ (ICC = 0.73; CV = 8.88%; SRD% = 22.43%) showed moderate agreement.

As quoted by Monark’s official web-site, “the value 0.0449 means that for each rpm magnet passing the rpm sensor t the crank rotated 0.0449 revolutions (14/52/6 = ratio flywheel to crank/6 magnets). The frequency of the reading is dependent on the rpm. With 100 rpm you have about (52/14 × 6 × 100/60) 37 data points per second and this is with the resolution of 57600Hz!” [36]. It can be said that it is currently possible to have more sensitive raw data at the end of the Wingate administrations, as is seen on technical information. However, the data flow speed at the beginning of the test may cause some problems. We had some problematic t@*v*_max_ and P@*v*_max_ outcomes which had negative values (i.e., −200 s), while others were valid and as expected. It seems that the ability of optical sensors may have failed to satisfactorily calculate t@*v*_max_ and P@*v*_max_ during high-speed movements of the flywheel. The rapid over-loading of the system during the acceleration phase may lead to a miscommunication between sensors and magnets. Note that we removed all test results of those participants who gave invalid data from our study. As of yet, to the authors' knowledge, there has been no study that has reported any findings related to t@*v*_max_ or P@*v*_max_ in the literature.

Since P@*v*_max_ is calculated based on a moment measured in milliseconds where the velocity is the highest, it is very sensitive. Similarly, t@*v*_max_ is also calculated based on instantaneous velocity data. Moreover, t_pp_, which is calculated as time to elicit peak power from start to middle of the one second period in milliseconds, gives a moderate level of reliability. However, other power indices are calculated by one second means of instantaneous raw data. Therefore, the calculated P@*v*_max_ is greater than the PP at the end of the Wingate all-out test due to differences in time periods considered.

Martin et al. [23] claim that their Power Cycle can measure power based on instantaneous data in every 3° of pedal crank rotation or averaged over one complete revolution of the crank. They have reported that calculated power with resistance provided solely by the moment of inertia of the flywheel was ~2100 watts and occurred at a pedaling rate of ~130 rpm, while power data averaged over one complete revolution of the pedal crank was ~1300 watts with ~120 rpm. Peak power outputs revealed in ~2 s, and mean values of athletes’ maximum velocity were reported as ~230–240 rpms as measured in a period less than ~3–4 s. It should be noted that Peak Bike’s flywheel-weight and inertial characteristics are exactly the same as the Power Cycle. Based on this information, it may be assumed that the start cadence of the Wingate all-out test has to be fixed around 120–130 rpms, and in this case, t_pp_ has to be revealed in a period of less than 2 s. Indeed, it is known that the most proper initial rpm to start the test is not the value of maximum crank velocities such as 230–240 rpms. Gullstrand and Larsson [18] suggest that “the power output is not optimal as the subject cannot pedal faster than up to a certain rate. Normally, most subjects will lose the neuromuscular controlled pedal frequency at approximately 200 rpm. This may occasionally be seen in an uneven frequency where for a short time the fluent pedaling is interrupted”. On the contrary, the value of 70 cadences shown as a default rpm in Monark software seems too low to reveal the highest individual peak power value. That is why we fixed the start rpm to the value of 120 based on the information obtained from Martin and his colleagues’ research [23]. In this context, the start rpm seems highly crucial. Instead of a fixed rpm, individually determined initial rpm may be a better alternative to improve new generation instantaneous power and time-related outcomes’ reliabilities; however, to the authors’ knowledge, there has not been a widely accepted method to evaluate a proper start rpm based on athletes’ individual performance characteristics.

Another important concept is the need for a comprehensive familiarization session. Since Peak Bike gives new generation power and time-related instantaneous measures, a comprehensive familiarization session that consists of (*a*) a session teaching overall pedaling technique, (*b*) short inertial-loading bursts without using any load, (*c*) a sufficient number of sprints with real test-load administrations, and (*d*) a whole all-out test practice for a 30-s test experience should be applied before real measurements are taken. Although many laboratories pay attention to athletes’ adaptations to test ergometer or protocol specificities when they test aerobic power (VO_2max_) or capacity, researchers do not spend enough time to familiarize the athletes if they test anaerobic power and/or capacity. On the contrary, anaerobic tasks can be more affected from lack of familiarization or non-standardized test administrations. Moreover, inattentive applications may alter instantaneous indices more dramatically than that of traditional ones. That is why it seems that a comprehensive familiarization session, which was performed in this study, is very important to increase determined test indices’ ICC values and to decrease the score of smallest worthwhile change (SWC).

Zajac et al. [37] indicated higher values of traditional power outputs in a 10-s Wingate administration with highly trained athletes. They also reported that peak power was reached more rapidly in 10 s than the 30-s test. They concluded that a psychological barrier in the 30-s test may affect the test’s anaerobic power outcomes. Since the instantaneous power outputs are more sensitive, they may be altered even more dramatically with the duration of the test. In order to examine power outputs related to explosive power such as PP, t_pp_, *v*_max_, etc., it may be more accurate to administer tests of shorter durations (i.e., 5 to 10 s) to participants.

Pekünlü and his colleagues [38] suggested a new calculation method for fatigue called the mechanical work drop (MWD). The MWD is calculated by integrating instantaneous raw power data. This integration method examines the area under the power time curve between the time at instantaneous peak power and the test ending. They reported that MWD (ICC = 0.92, CV = 4.53%) may be more sensitive to the detection of real changes in fatigue compared to FI (ICC = 0.71, CV = 6.46%). In the present study, the calculated mechanical work drop’s ICC correlation was found to be 0.98 (CV = 2.31%) which is still greater than the PD and FI%. 

Mean *e*_tot_ scores obtained from Peak Bike’s software were approximately 20,000–21,000 joules in the present study. However, the total metabolic energy expenditure of the Wingate all-out test was previously calculated by analyzing the fractions of the energy from aerobic, anaerobic alactic, and lactic acid metabolisms [39,40]. It was shown that a total of ~125–130 kJ of energy is utilized during a standard 30-s all-out test, which is highly above than the *e*_tot_ that was seen in the present study, likely because this value refers to an indirect measure of work done throughout the 30 s but not a value of metabolic energy expenditure. The value of *e*_tot_ indirectly calculated based on raw data was seen to be highly reliable (ICC = 0.97 and CV = 1.74%).

Surprisingly, average power indices calculated by traditionally based 5-s means (AP_5s_ = ~720 W) were found to be significantly lower than that of calculated based on instantaneous raw data (AP = ~760 W) (*p* < 0.01). This may have resulted from inertial correction settings of the software used. The underlying reasons for the differences should be clarified by the Wingate Institute.

## 5. Conclusions

Reliability results of traditional power indices calculated based on 5-s means were similar with the previous findings in literature. Instantaneous new generation power indices are also reliable test outcomes. However, t_pp_, t@*v*_max_, and P@*v*_max_, which are calculated based on instantaneous raw data, are more sensitive than the others. This may be because it seems that they reflect a moment where the rpm is the highest, while the others are calculated by one second means. SRDs% for t_pp_, t@*v*_max_, and P@*v*_max_ were between 12.46 and 22.43, which was considered to be low [41]. Operators may have some nonsense t@*v*_max_ and P@*v*_max_ data at the end of some tests. We inferred that this problem could be caused by a miscommunication in data flow during high-speed movements of the flywheel. Test administers should consider the fact that new generation power indices could be more easily affected by a lack of familiarization.

## Figures and Tables

**Table 1:** Results of test-retest reliability of traditional power indices calculated based on 5-s means (*n* = 32).

Variable	Session 1	Session 2	ICC (95% CL)	Cohen’s d	SEM	SRD	SRD%	CV (95% CL)	SWC
PP_5s_ (W)	1060.33 ± 122.70	1065.92 ± 127.32	0.987 (0.974–0.994)	−0.05	9.95	0.28	2.69	0.97 (0.45–1.49)	2.81
AP_5s_ (W)	710.90 ± 82.41	728.02 ± 88.83	0.984 (0.967–0.992)	−0.20	11.04	0.31	4.27	1.54 (0.58–2.12)	3.12
LP_5s_ (W)	457.69 ± 74.29	472.52 ± 77.37	0.966 (0.931–0.984)	−0.20	12.28	0.34	7.66	2.77 (1.56–3.97)	3.47
PD_5s_ (W·s^−1^)	20.09 ± 3.63	19.78 ± 3.29	0.970 (0.938–0.985)	0.09	0.45	0.01	6.38	2.30 (1.29–3.31)	0.13
FI_5s_ (%)	56.63 ± 6.53	55.59 ± 5.79	0.948 (0.893–0.975)	0.17	1.08	0.03	5.23	1.89 (1.05–2.72)	0.30

PP_5s_ = Peak power in 5-s means; AP_5s_ = Average power in 5-s means; LP_5s_ = Lowest power in 5-s means; PD_5s_ = Power drop based on 5-s means; FI_5s_ (%) = Fatigue index ratio in 5-s means; ICC = Intra-class correlation coefficient; SEM = Standard error of measurement; SRD = Smallest real difference; CV = Coefficient of variation; SWC = Smallest worthwhile change; Data are means ± SD.

**Table 2:** Results of test-retest reliability of new generation power indices (*n* = 32).

Variable	Session-1	Session-2	ICC (95% CL)	Cohen’s d	SEM	SRD	SRD%	CV (95% CL)	SWC
PP (W)	1169.35 ± 142.57	1165.15 ± 142.16	0.986 (0.972–0.993)	−0.03	11.82	0.33	2.89	1.04 (0.52–1.56)	3.34
t_PP_ (s)	1.17 ± 0.16	1.23 ± 0.20	0.730 (0.447–0.868)	−0.33	0.06	0.01	12.56	4.53 (2.07–7.00)	0.02
AP (W)	760.54 ± 84.68	764.41 ± 92.55	0.988 (0.976–0.994)	−0.04	6.97	0.19	2.48	0.89 (0.48–1.31)	1.97
LP (W)	401.46 ± 74.42	403.45 ± 73.02	0.944 (0.886–0.973)	−0.03	12.00	0.33	8.78	4.28 (0.74–7.81)	4.52
PD (W·s^−1^)	25.60 ± 4.47	25.08 ± 4.01	0.973 (0.945–0.987)	0.12	0.53	0.01	5.92	3.21 (−0.07–6.49)	0.24
FI (%)	65.43 ± 6.41	64.48 ± 5.63	0.938 (0.873–0.970)	0.15	1.09	0.03	4.64	2.82 (−0.52–6.15)	0.07
*v*_max_ (rpm)	148.54 ± 5.59	148.55 ± 5.71	0.957 (0.911–0.979)	−0.01	0.76	0.01	1.42	1.68 (−1.69–5.05)	0.70
P@*v*_max_ (W)	1240.90 ± 180.46	1238.16 ± 184.42	0.755 (0.498–0.880)	0.02	63.01	1.75	14.63	6.55 (2.81–10.28)	22.74
t@*v*_max_ (ms)	2.30 ± 0.51	2.31 ± 0.61	0.730 (0.508–0.883)	−0.02	0.18	0.01	22.43	8.88 (3.89–13.86)	0.06
P_dec_ (W)	747.33 ± 138.10	740.86 ± 123.59	0.967 (0.932–0.984)	0.05	17.30	0.48	6.41	3.43 (0.05–6.81)	3.43
*e*_tot_ (kJ)	21.47 ± 2.51	21.33 ± 2.73	0.968 (0.934–0.984)	0.05	0.37	10.36	4.81	1.74 (1.15–2.33)	0.11

PP = Peak power; AP = Average power; LP = Lowest power; PD = Power drop; FI (%) = Fatigue index ratio; t_PP_ = Time to peak power; *v*_max_ = Maximum velocity; P@*v*_max_ = Power at maximum speed; t@*v*_max_ = Time at maximum speed; P_dec_ = Decline in power; *e*_tot_ = Total energy expenditure; ICC = Intra-class correlation coefficient; SEM = Standard error of measurement; SRD = Smallest real difference; CV = Coefficient of variation; SWC = Smallest worthwhile change; Data are means ± SD.
